# Cardiomyocyte-specific disruption of Cathepsin K protects against doxorubicin-induced cardiotoxicity

**DOI:** 10.1038/s41419-018-0727-2

**Published:** 2018-06-07

**Authors:** Rui Guo, Yinan Hua, Jun Ren, Karin E. Bornfeldt, Sreejayan Nair

**Affiliations:** 1 Center for Cardiovascular Research and Alternative Medicine, School of Pharmacy College of Health Sciences, University of Wyoming, Laramie, WY 82071 USA; 2 UW Medicine Diabetes Institute, Departments of Medicine, Division of Metabolism, Endocrinology and Nutrition, and Pathology, School of Medicine, University of Washington, Seattle, WA 98109 USA

## Abstract

The lysosomal cysteine protease Cathepsin K is elevated in humans and animal models of heart failure. Our recent studies show that whole-body deletion of Cathepsin K protects mice against cardiac dysfunction. Whether this is attributable to a direct effect on cardiomyocytes or is a consequence of the global metabolic alterations associated with Cathepsin K deletion is unknown. To determine the role of Cathepsin K in cardiomyocytes, we developed a cardiomyocyte-specific Cathepsin K-deficient mouse model and tested the hypothesis that ablation of Cathepsin K in cardiomyocytes would ameliorate the cardiotoxic side-effects of the anticancer drug doxorubicin. We used an α-myosin heavy chain promoter to drive expression of Cre, which resulted in over 80% reduction in protein and mRNA levels of cardiac Cathepsin K at baseline. Four-month-old control (Myh-Cre^-^; *Ctsk* ^fl/fl^) and Cathepsin K knockout (Myh-Cre^+^; *Ctsk* ^fl/fl^) mice received intraperitoneal injections of doxorubicin or vehicle, 1 week following which, body and tissue weight, echocardiographic properties, cardiomyocyte contractile function and Ca^2+^-handling were evaluated. Control mice treated with doxorubicin exhibited a marked increase in cardiac Cathepsin K, which was associated with an impairment in cardiac structure and function, evidenced as an increase in end-systolic and end-diastolic diameters, decreased fractional shortening and wall thickness, disruption in cardiac sarcomere and microfilaments and impaired intracellular Ca^2+^ homeostasis. In contrast, the aforementioned cardiotoxic effects of doxorubicin were attenuated or reversed in mice lacking cardiac Cathepsin K. Mechanistically, Cathepsin K-deficiency reconciled the disturbance in cardiac energy homeostasis and attenuated NF-κB signaling and apoptosis to ameliorate doxorubicin-induced cardiotoxicity. Cathepsin K may represent a viable drug target to treat cardiac disease.

## Introduction

Cathepsin K is a lysosomal cysteine protease, which has been widely studied in the context of osteoporosis^1^. Recently, reports suggest that activation of this protease may have a deleterious role in the pathophysiology of cardiometabolic diseases^2–4^. Elevated levels of Cathepsin K have been demonstrated in both human and animal models of heart failure, atherosclerosis, and coronary heart disease^5–7^. Studies from our laboratory have revealed that global deletion of the Cathepsin K gene protects against cardiac dysfunction associated with pressure overload, diabetes, and aging^8–11^. However, it is unclear whether the beneficial effects of Cathepsin K knockout is attributable to its direct cardiac effects or a secondary effect consequent to improvement in whole-body metabolic changes. To determine whether Cathepsin K has a direct role in cardioprotection, we generated a conditional, cardiomyocyte-specific, Cathepsin K knockout mouse model.

Doxorubicin is a potent and effective broad-spectrum antineoplastic anthracycline antibiotic used to treat a variety of cancers. However, the clinical use of doxorubicin is limited by its cardiotoxicity that leads to cardiomyopathy and heart failure. Doxorubicin-induced cardiotoxicity is cumulative and irreversible, and manifests as unfavorable cardiac morphologic and functional changes, including dilated cardiac chambers, impaired left ventricular contractility, reduced ejection fraction, reduced cardiac output, and associated diastolic dysfunction, as well as histopathologic alterations^12^. Previous studies have shown that doxorubicin-induced cardiotoxicity can be attributed to intracellular calcium disturbance, nucleic interruption and damage, impaired cardiac energy homeostasis, apoptosis, and oxidative stress resulting either from increased levels of reactive oxygen species and lipid peroxidation or reduced levels of antioxidants and sulfhydryl groups^13–15^.

In this study, we evaluated the effects of cardiomyocyte-specific Cathepsin K-deficiency on doxorubicin-induced cardiac dysfunction. To understand the molecular mechanisms, we also evaluated the metabolic, apoptotic and inflammatory signaling.

## Results

### Generation and characterization of cardiac-specific Cathepsin K knockout (*Ctsk*-CKO) mice

**Fig. 1:**
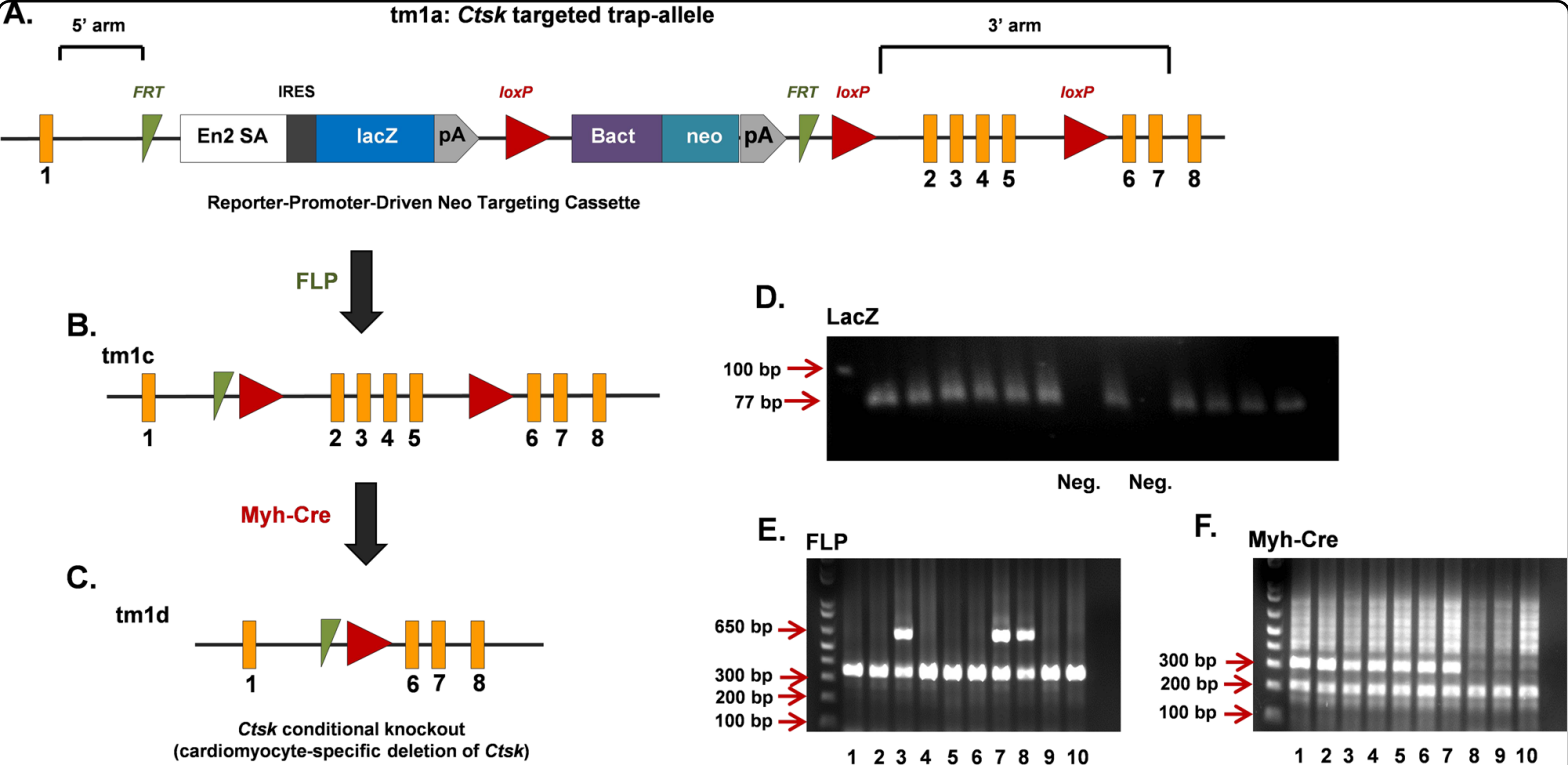
Generation and genotyping of cardiac-specific *Ctsk*-CKO mice. **a** tm1a allele: representing targeted *Ctsk* allele containing LacZ reporter-promoter-driven neo targeting cassette, FLP-*FRT* sites, Cre-*loxP* sites and *Ctsk* exons (numbered 1–8); **b** tm1c allele: the conditional allele representing the “floxed” mice generated by crossing “tm1a” mice to FLP recombinase transgenic mice; **c** tm1d allele: the null allele representing the cardiac-specific *Ctsk*-conditional knockout mice generated by crossing “tm1c” (floxed) mice to Myh-Cre recombinase transgenic mice. The *FRT* sites are indicated by green triangles, the *loxP* sites are represented by red triangles, the exons are represented by orange rectangles. **d** Representative image of LacZ genotyping: LacZ positive targeted band is 77 bp; LacZ negative mice display no bands. **e** Representative image of FLP genotyping: mutant-band is 340 bp; wild-type band is 650 bp; heterozygotes have both bands; homozygotes only have bands at 340 bp; mice have mutant bands are FLP positive. **f** Representative image of Myh-Cre genotyping: mutant-band is 300 bp; internal control band is 200 bp; mice have mutant bands are Myh-Cre positive. Both Myh-Cre positive and FLP positive are cardiac-specific *Ctsk*-CKO mice. Mice 1, 2, 4, 5, and 6 are used as cardiac-specific *Ctsk*-CKO (Myh-Cre^+^; *Ctsk*^fl/fl^); mice 9 ~ 10 are used as control (Myh-Cre^−^; *Ctsk*^fl/fl^)

Mice with cardiomyocyte-targeted deletion of Cathepsin K were generated by utilizing ES cells carrying *FRT* and *loxP* sites flanking *Ctsk* exon 2–5 that encodes the active site of Cathepsin K (Fig. 1a). Expression of Cre recombinase under control of the myh6 promoter results in deletion of exons 2–5 of *Ctsk* in the cardiomyocytes (Fig. 1b, c). Genotyping showed a LacZ positive band of 77 bp (Fig. 1d); FLP mutant band of 340 bp, wild-type band of 650 bp (Fig. 1e); and Myh-Cre mutant band of 300 bp with an internal control band of 200 bp (Fig. 1f). *Ctsk* mRNA levels were analyzed in left ventricles of control and *Ctsk* CKO mice using quantitative real-time PCR. Levels of *Ctsk* mRNA were reduced by 86.5% using primers targeting exon 2 and by 84.2% using primers targeting its coding region in *Ctsk*-CKO mice, as compared with littermate controls (Supplementary Figure 1A-B). Western blot analysis confirmed reduction in Cathepsin K protein levels in hearts from *Ctsk*-CKO mice, as compared with littermate control mice (Supplementary Figure 1C-D). There were no significant differences in the levels of Cathepsin K between wild type (WT) and control mice (Supplementary Figure 1E-F).

### General biometric and echocardiographic properties

**Fig. 2:**
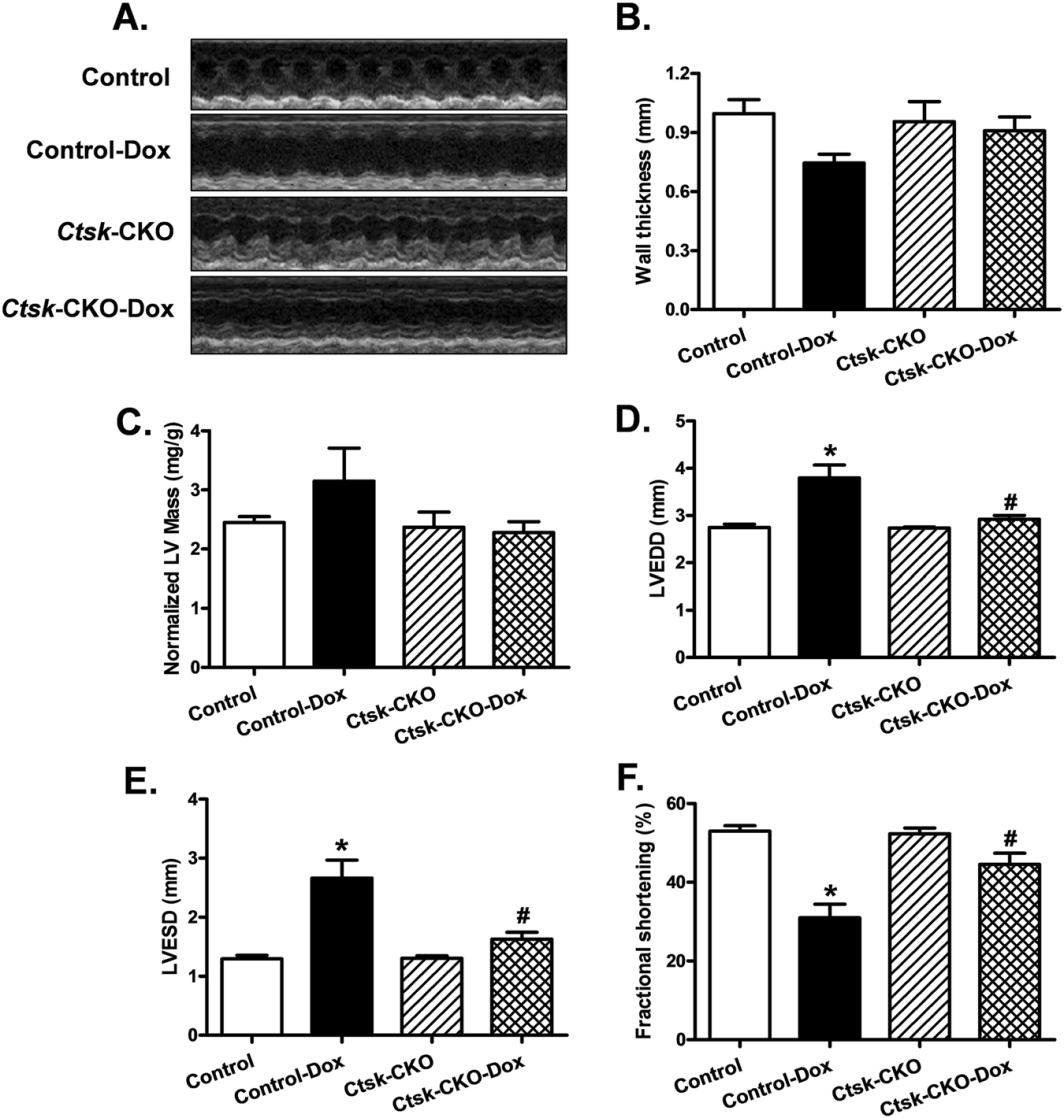
Echocardiographic properties in control and cardiac-specific *Ctsk*-CKO mice treated with or without doxorubicin. **a** Representative echocardiographic images; **b** Wall thickness; **c** Left ventricular (LV) mass normalized to body weight; **d** LV end-diastolic diameter; **e** LV end-systolic diameter; **f** Fractional shortening. Mean ± SEM, *n* = 6–8 mice per group. *p < 0.05 vs. Control group, #*p* < 0.05 vs. Control-Dox group

Doxorubicin treatment resulted in decreased body weight gain in both control and *Ctsk*-CKO mice, without significant alterations in their heart mass (Supplementary Figure 2). Representative echocardiographic images are shown in Fig. 2a. The wall thickness and normalized LV mass were comparable among control and *Ctsk*-CKO mice in both vehicle and doxorubicin-treated groups (Fig. 2b, c). However, treatment with doxorubicin significantly increased left ventricular end-diastolic dimension (LVEDD) and left ventricular end-systolic dimension (LVESD) in the control mice but not in the *Ctsk*-CKO mice (Fig. 2d, e). Furthermore, cardiomyocyte-specific *Ctsk* deficiency reconciled doxorubicin-induced depressed fractional shortening (Fig. 2f).

### Cardiomyocyte contractile function and intracellular Ca^2+^ properties

**Fig. 3:**
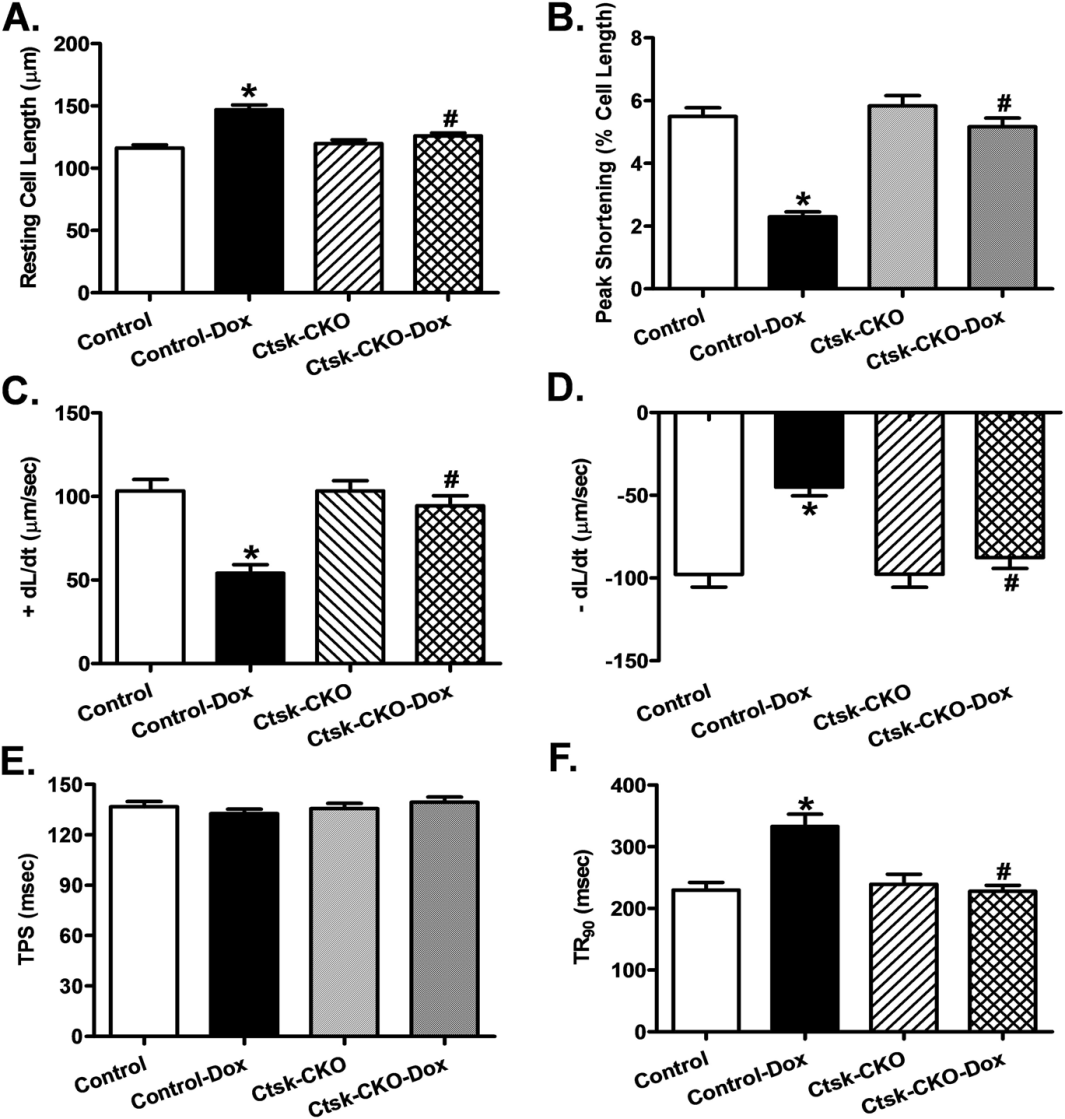
Cardiomyocyte contractile properties in control and cardiac-specific *Ctsk*-CKO mice treated with or without doxorubicin. **a** Resting cell length; **b** Peak shortening (PS), normalized to cell length; **c** Maximal velocity of shortening (+dL/dt); **d** Maximal velocity of relengthening (−dL/dt); **e** Time-to PS (TPS); **f** Time-to-90% relengthening (TR90). Mean ± SEM, *n* = 88–102 cells from three to four mice per group. *p < 0.05 vs. Control group, #*p* < 0.05 vs. Control-Dox group

**Fig. 4:**
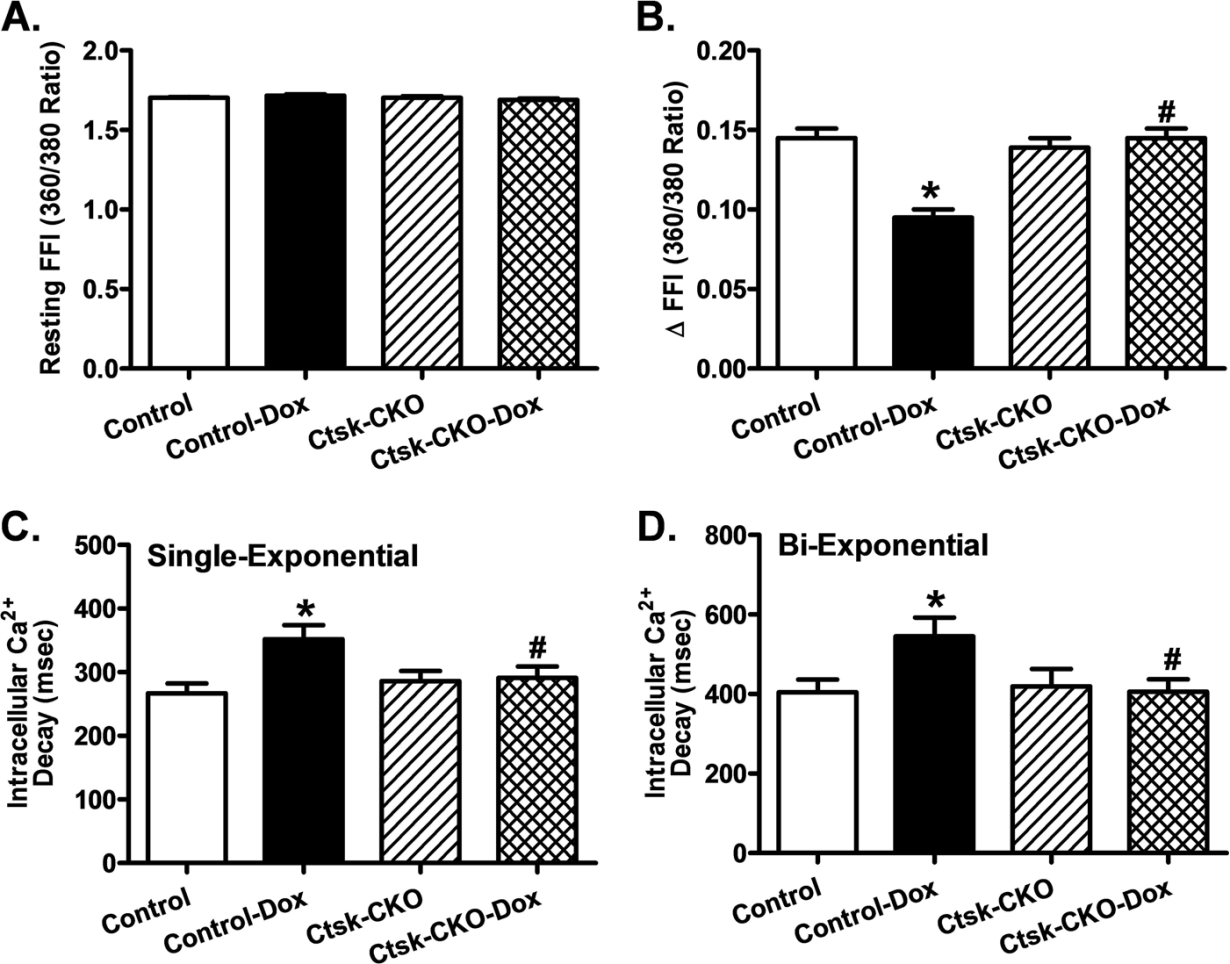
Cardiomyocyte intracellular Ca^2+^ handling properties in control and cardiac-specific *Ctsk*-CKO mice treated with or without doxorubicin. **a** Resting fura-2 fluorescence intensity (FFI); **b** Electrically stimulated rise in FFI (ΔFFI); **c** Intracellular Ca^2+^ decay rate (single exponential); **d** Intracellular Ca^2+^ decay rate (bi-exponential). Mean ± SEM, *n* = 80–96 cells from three to four mice per group. *p < 0.05 vs. Control group, #*p* < 0.05 vs. Control-Dox group

As depicted in Fig. 3a, cardiac-specific Cathepsin K-deficiency prevented the increase in resting cardiomyocyte length induced by doxorubicin. However, cardiomyocytes isolated from control mice challenged with doxorubicin exhibited a significantly reduced peak shortening (PS) and maximal velocity of shortening/relengthening (±dL/dt), as well as prolonged time-to-90% relengthening (TR90), all of which were markedly diminished in cardiomyocytes isolated from *Ctsk*-CKO mice treated with doxorubicin (Fig. 3b–d,f). There was no significant difference in time-to-peak shortening (TPS) in isolated cardiomyocytes between control and *Ctsk*-CKO mice, with or without doxorubicin treatment (Fig. 3e). In addition, data presented in Fig. 4 reveals a significantly depressed intracellular Ca^2+^ rise in response to electrical stimulus (ΔFFI) (Fig. 4b), reduced intracellular Ca^2+^ decay rate (single- and Bi-exponential curve fit) (Fig. 4c, d), and an unchanged resting intracellular Ca^2+^ concentration (Fig. 4a) in cardiomyocytes isolated from doxorubicin-treated control mice compared to those isolated from vehicle-treated mice. Cardiac-specific Cathepsin K-deficiency negated doxorubicin-induced prolongation in intracellular Ca^2+^ decay and depression in ΔFFI without affecting the baseline FFI. Furthermore, Cathepsin K inhibitor-II reconciled doxorubicin-induced myocyte contractile anomalies including reduced PS and ±dL/dt, as well as prolonged TR_90_ (Supplementary Figure 4). Additionally, this effect was blunted by the pre-treatment of the cardiomyocytes with apoptosis activator II or the AMPK activator AICAR. On the other hand, inhibition NF-κB using PDTC partially attenuated doxorubicin-triggered abnormal cardiomyocyte contractile function. TPS was not significantly affected by any of the drug treatment.

### Cardiomyocyte cross-sectional area, fibrosis, and myofibrillar disruption

**Fig. 5:**
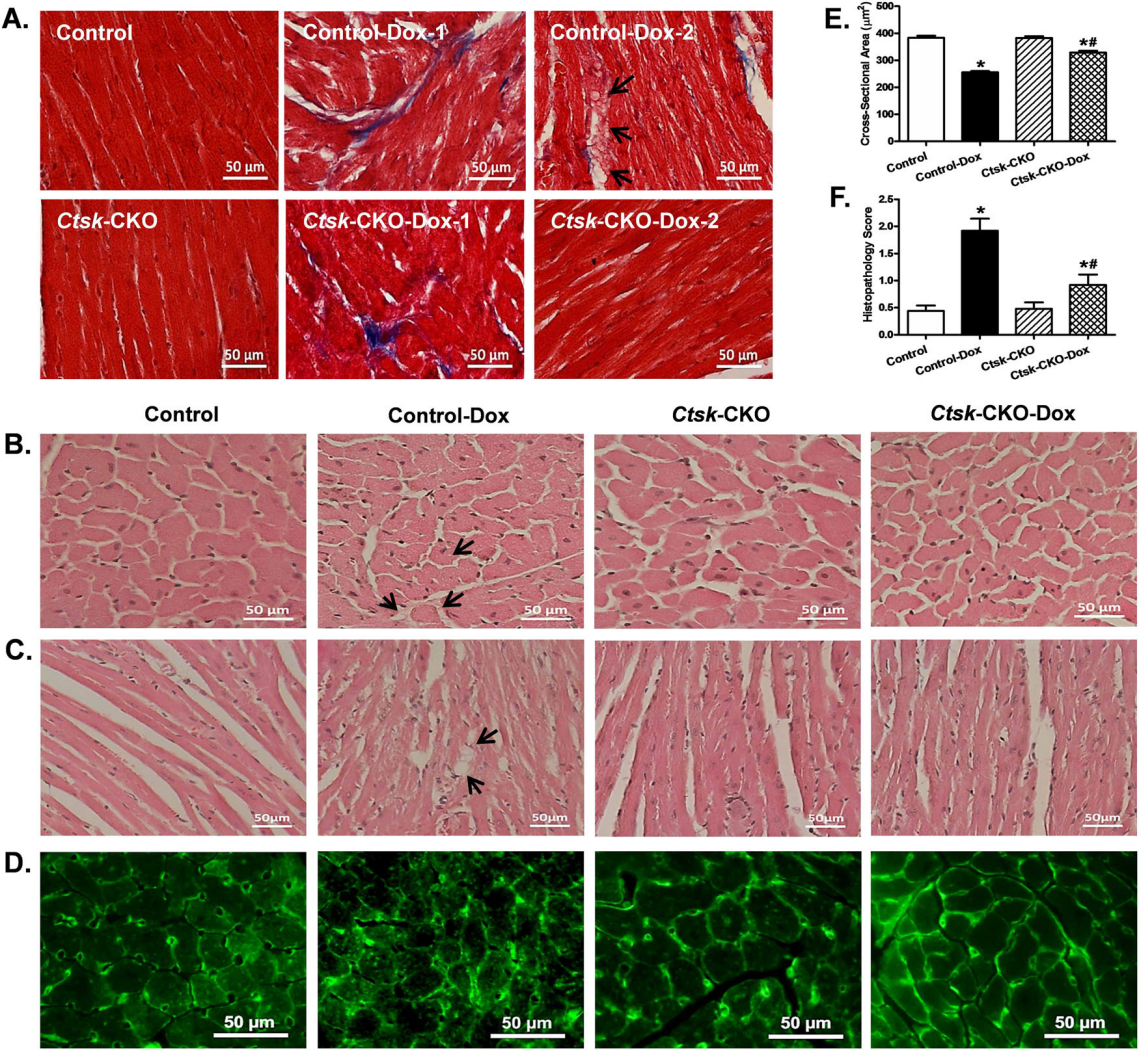
Histological analyses in hearts from control and cardiac-specific *Ctsk*-CKO mice treated with or without doxorubicin. **a** Representative images of Masson trichrome staining for fibrosis (×400; scale bar = 50 μm); **b** Representative H&E staining micrographs of transverse sections of left ventricular myocardium (×400; scale bar = 50 μm); **c** Representative H&E staining micrographs of longitudinal sections of left ventricular myocardium (×200; scale bar = 50 μm); **d** Representative Lectin staining of transverse sections of left ventricular myocardium (×400; scale bar = 50 μm); **e** Quantitative cardiomyocyte cross-sectional (transverse) area using measurements of 230 cardiomyocytes from three mice per group; **f** Semi-quantitative vacuolization and myofibrillar degeneration of the cardiomyocytes by scoring scales from 25 slides per group. Mean ± SEM, *p < 0.05 vs. Control group, #*p* < 0.05 vs. Control-Dox group. Arrow: cytoplasmic vacuolization and myofibrillar degeneration

**Fig. 6:**
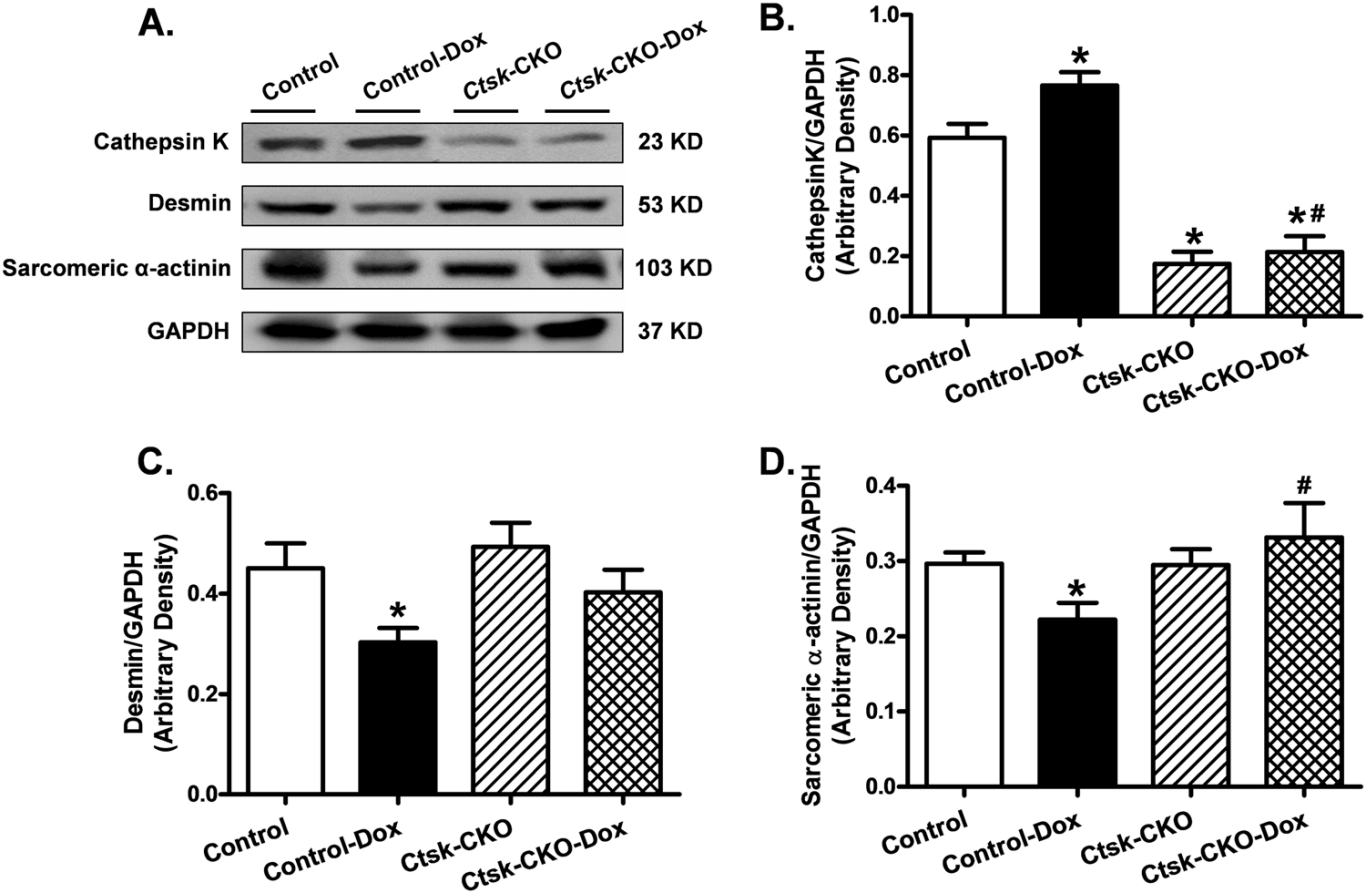
Western blot analysis exhibiting levels of Cathepsin K and myofibrillar protein markers in myocardium from control and cardiac-specific *Ctsk*-CKO mice treated with or without doxorubicin. **a** Representative gel blots depicting expressions of Cathepsin K, desmin, sarcomeric α-actinin, and GAPDH (loading control); **b** Cathepsin K/GAPDH; **c** Desmin/GAPDH; **d** Sarcomeric α-actinin/GAPDH. Mean ± SEM, *n* = 6–8 mice per group, *p < 0.05 vs. Control group, #*p* < 0.05 vs. Control-Dox group

To evaluate the impact of *Ctsk*-CKO on myocardial histology in doxorubicin-treated mice, cardiomyocyte cross-sectional area, myofibrillar disruption and cardiac fibrosis were examined (Fig. 5). Masson trichrome staining revealed the presence of cardiac fibrosis in the hearts of both control and *Ctsk*-CKO mice treated with doxorubicin; whereas, myofibrillar disruption was seen only in doxorubicin-treated control mice (Fig. 5a). Hematoxylin and eosin staining and lectin staining of the histologic sections showed decreased cardiomyocyte cross-sectional area, cytoplasmic vacuolization and myocytolysis (myofibrillar disruption) following doxorubicin treatment. In contrast, cardiomyocytes from *Ctsk*-CKO mice subjected to doxorubicin did not exhibit these anomalies (Fig. 5b–e). Semi-quantitative analysis of myocardial vacuolization and myofibrillar degeneration is provided in Fig. 5f. Next, sarcomeric cytoskeletal proteins, including desmin and α-actinin, as well as Cathepsin K in the myocardium were determined by western blot (Fig. 6). Doxorubicin challenge upregulated the expression of Cathepsin K protein while downregulating desmin and sarcomeric α-actinin. Additionally, doxorubicin-induced attenuation of desmin and sarcomeric α-actinin were negated in *Ctsk*-CKO mice.

### Cardiac apoptosis, inflammation, metabolic and survival signaling

**Fig. 7:**
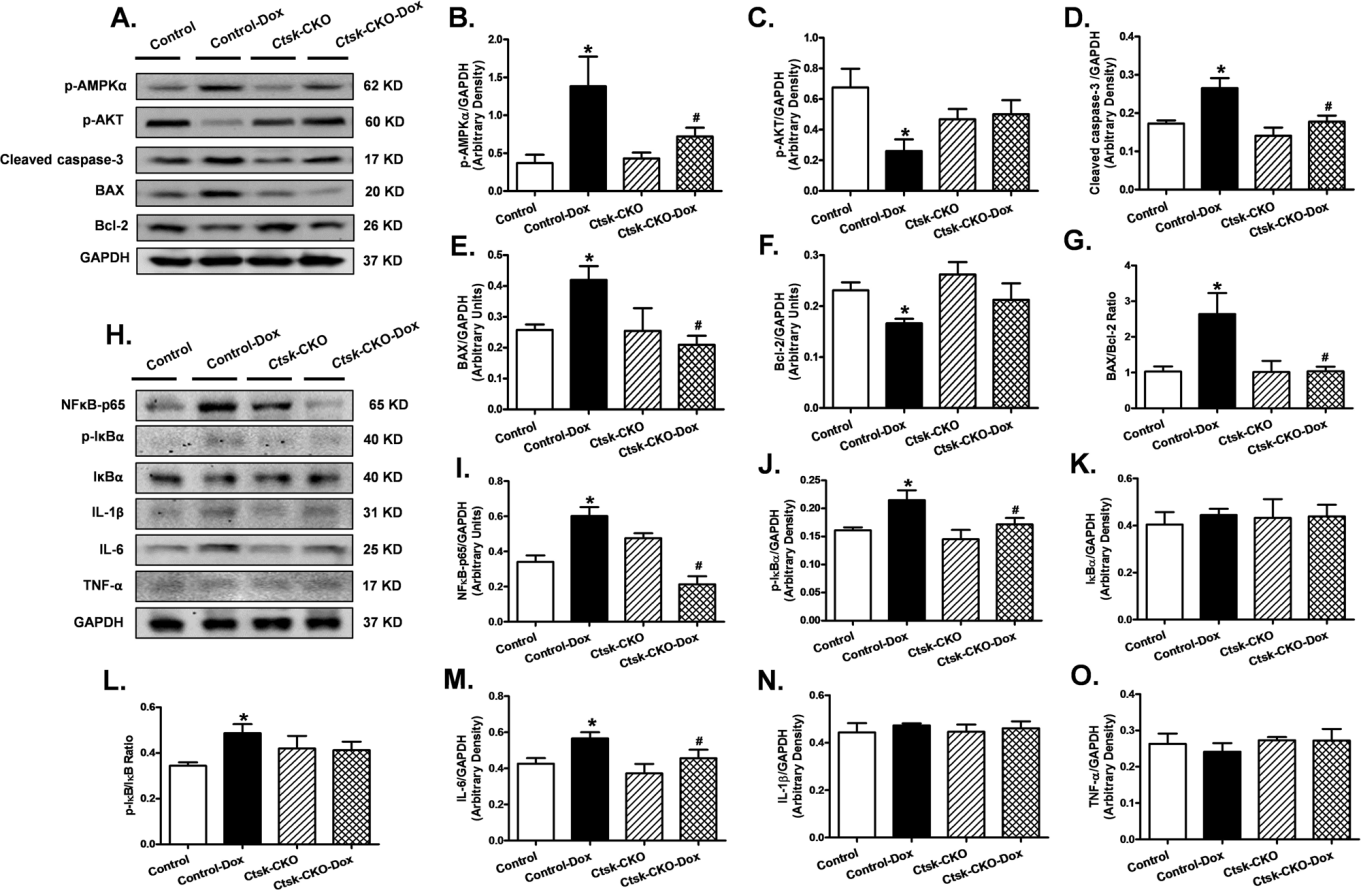
Western blot analysis exhibiting levels of metabolic and survival signaling, apoptotic markers, and inflammatory markers in myocardium from control and cardiac-specific *Ctsk*-CKO mice treated with or without doxorubicin. **a** Representative gel blots depicting expressions of p-AMPKα, p-AKT, cleaved caspase-3, BAX, Bcl-2, and GAPDH (loading control); **b** p-AMPKα/GAPDH; **c** p-AKT/GAPDH; **d** Cleaved-caspase-3/GAPDH; **e** BAX/GAPDH; **f** Bcl-2/GAPDH; **g** BAX/Bcl-2 ratio; **h** Representative gel blots depicting expressions of NFκB-p65, phosphorylated IκBα, IκBα, IL-1β, IL-6, TNF-α, and GAPDH (loading control); **i** NFκB-p65/GAPDH; **j** **p**-IκBα/GAPDH; **k** IκBα/GAPDH; **l** p-IκBα/IκBα ratio; **m** IL-6/GAPDH; **n** IL-1β/GAPDH; **o** TNF- α/GAPDH. Mean ± SEM, *n* = 6–8 mice per group, *p < 0.05 vs. Control group, #*p* < 0.05 vs. Control-Dox group

**Fig. 8:**
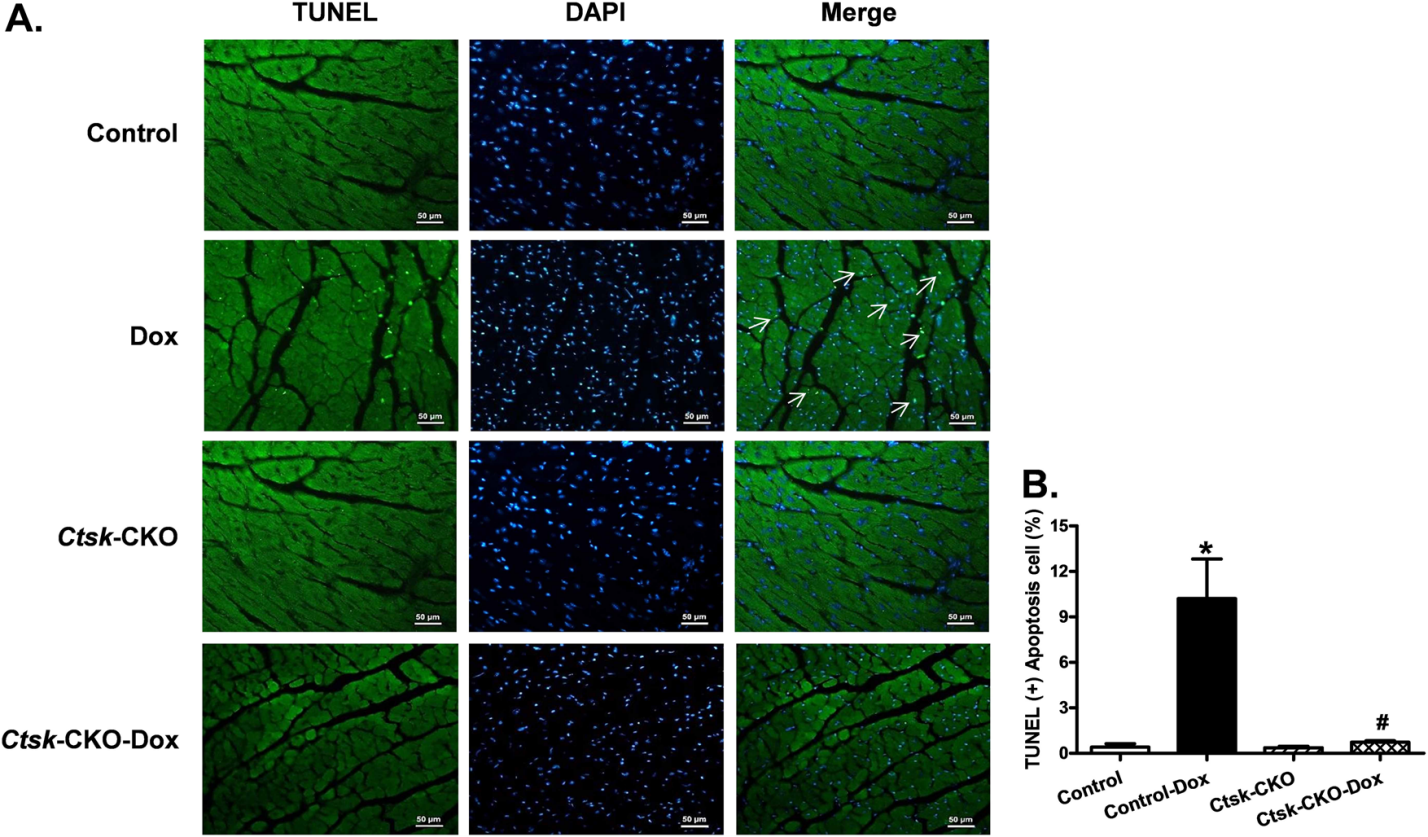
TUNEL staining of apoptosis in myocardium from control and cardiac-specific *Ctsk*-CKO mice treated with or without doxorubicin. **a** Representative photomicrograph of the TUNEL staining; **b** Quantitative analysis of the apoptotic cells. Mean ± SEM, *p < 0.05 vs. Control group, #*p* < 0.05 vs. Control-Dox group. TUNEL-positive nuclei were visualized with green fluorescein. All nuclei were stained with DAPI shown in blue color. Apoptotic cells are indicated by white arrows

Because doxorubicin has been shown to mediate its deleterious effects via inflammation and cardiac apoptosis, as well as the disturbance of cardiac energy metabolism, we next determined the effect of *Ctsk*-CKO on cardiac apoptosis, inflammation, metabolic, and survival signaling in the myocardium. To this end, we measured the phosphorylation levels of metabolic regulators AKT and AMPKα, pro-apoptotic markers BAX and cleaved caspase-3, anti-apoptotic marker Bcl-2, as well as mediators of inflammation, including NFκB-p65, p-IκBα, IκBα, IL-1β, IL-6, and TNF-α using western blot analysis. Moreover, glycolytic and oxidative flux was measured by determining the formation of cardiac lactate from pyruvate. Our data revealed that doxorubicin treatment increased levels of p-AMPKα, BAX, cleaved caspase-3, NFκB-p65, p-IκBα, and IL-6 and dampened p-AKT and Bcl-2, all of which were mitigated in the *Ctsk*-CKO mice. There were no significant changes in protein expression of IκBα, IL-1β, and TNF-α with either the knockout or doxorubicin challenge (Fig. 7). Cardiac lactate production was elevated in doxorubicin-treated control mice, which was reversed in *Ctsk*-CKO mice (Supplementary Figure 3). To further ascertain changes in apoptosis, we performed TUNEL staining on our tissue sections. The TUNEL-positive nuclei visualized in fluorescein green as a percentage of all nuclei stained with DAPI (blue) were significantly higher in the myocardium from doxorubicin-treated control mice. In contrast, we observed fewer TUNEL-positive nuclei in the knockout mice subjected to doxorubicin. Knockout of *Ctsk* by itself did not affect the TUNEL-positive nuclei in the absence of doxorubicin treatment (Fig. 8).

## Discussion

Our study demonstrated that cardiomyocyte-specific knockout of Cathepsin K mitigates doxorubicin-induced cardiac structural and functional defects, suggesting a direct involvement of Cathepsin K in cardiotoxicity mediated by doxorubicin. Here we provide the evidence that cardiomyocyte-specific deletion of Cathepsin K attenuated or ablated doxorubicin-induced aberrant cardiac structure and function, abnormal cardiomyocyte morphology, myofibrillar degeneration, impaired energy metabolism, inflammatory response, and apoptosis. These findings not only support our previous studies that global knockout of Cathepsin K reconciled streptozotocin-, high-fat diet-, and pressure overload-induced cardiac dysfunction^8, 9, 11^, but extend these findings to show that the deleterious effects of Cathepsin K in these models are, at least in part, attributable to its cardiac-specific effects. The protective effects of Cathepsin K ablation were associated with alterations in multiple signaling pathways, including restoration of phosphorylation of AMPK, reduction in pro-apoptotic proteins BAX and cleaved-caspase-3, blunted NF-κB signaling, as well as recovery of phosphorylation of AKT.

Previous studies have reported that Cathepsin K levels were elevated in the hearts of human subjects and experimental animals with dilated cardiomyopathy, hypertrophic cardiomyopathy, and heart failure^5, 8, 11, 16^. While our current study confirms the upregulation of Cathepsin K expression in myopathic heart following doxorubicin treatment, it also supports the notion that Cathepsin K upregulation may be a cause rather than consequence of the disease. Consistent with previous studies, doxorubicin administrated resulted in enlargement of left ventricular chamber assessed as increased LVESD and LVEDD, as well as reduced fractional shortening in control hearts, reminiscent of cardiac geometric changes associated with dilated cardiomyopathy and cardiac remolding^12, 17–19^. These changes were dramatically attenuated in *Ctsk*-CKO mice, indicating that the absence of Cathepsin K in the myocardium may protect against cardiac remodeling and dysfunction. Moreover, data from this study showed that *Ctsk*-CKO reconciled doxorubicin-induced cardiomyocyte contractile dysfunction and disturbed intracellular Ca^2+^ homeostasis manifested as suppressed peak shortening and maximal velocity of shortening/relengthening, prolonged time-to-90% relengthening, as well as depressed electrically stimulated rise and clearance in intracellular Ca^2+^, which is consistent with our previous work using global Cathepsin K knockout mice^8, 11^. The length of single cardiomyocyte was significantly increased; whereas, the cross-sectional area of the cardiomyocytes was remarkably decreased by doxorubicin treatment. Furthermore, doxorubicin caused a severe cardiac cytoplasmic vacuolization and myofibrillar disruption with a loss-of-desmin and sarcomeric α-actinin expression in control mice. All these effects were markedly ameliorated by cardiac-specific deletion of Cathepsin K, suggesting a critical role of cardiac Cathepsin K in myofibrillar remodeling and cardiomyocyte injury, contributing to the doxorubicin-induced whole heart structure and functional changes that are similar to dilated cardiomyopathy.

The impaired myofibrils and myocyte vacuolization as typical features of doxorubicin cardiotoxicity have been observed in several previous studies^12, 19–22^, and the mutation or deletion of cytoskeletal proteins, such as α-actinin and desmin have been shown to contribute to dilated cardiomyopathy^23, 24^. Desmin, as a major intermediate filament cytoskeleton, makes the organization of myofibrils and maintains the structural and mechanical integrity of cardiomyocytes^25^. Loss-of-desmin was detected at the end stage of heart failure^26^. Mice lacking desmin were found to develop dilated cardiomyopathy, smooth muscle, and skeletal muscle defects^25, 27, 28^. Similarly, α-actinin localized at the Z-disk is a cytoskeletal actin-binding protein, which also plays important structural and regulatory roles in cytoskeletal arrangement and muscle contraction^29, 30^. Defects in sarcomeric α-actinin (ACTN2) can cause both hypertrophic and dilated cardiomyopathy^31, 32^. These cytoskeletal defects may be associated with apoptosis, and act as compensatory responses to apoptotic activation, leading to cardiomyocyte contractile dysfunction before cell death^33–35^. Other studies also have shown that caspase-3 is able to cleave α-actin and desmin, resulting in an impaired Ca^2+^-activated force and myofibrillar ATPase activity, and promoting apoptosis^35, 36^. Our present study revealed a significant enhancement of apoptosis in the heart following doxorubicin treatment as evidenced by an increase in the protein levels of cleaved-caspase-3 and BAX, and decreased Bcl-2, accompanied with reduced desmin and sarcomeric α-actinin, as well as upregulation of Cathepsin K, all of which were attenuated in cardiac Cathepsin K knockout mice. These results indicate that the deletion of Cathepsin K in the cardiomyocyte prevents doxorubicin-activated apoptosis and subsequent myofibrillar degeneration and contractile dysfunction. Other proteases such as calpain and Cathepsin B have been reported to cleave desmin, which may also be a substrate for Cathepsin K^37^. Interestingly, the absence of cardiac Cathepsin K had no effect on doxorubicin-induced myocardial fibrosis, suggesting that the protective role of *Ctsk*-CKO against doxorubicin toxicity may be independent of extracellular matrix remodeling.

In this study, we also demonstrated that cardiomyocyte-specific Cathepsin K knockout alleviated doxorubicin-induced AMPK and NF-κB activation in cardiac tissue, and restored phosphorylation of AKT, which are blunted following doxorubicin treatment. Accumulating evidence indicates a vital role of perturbation of energy metabolism and mitochondrial dysfunction in cardiac pathological responses to doxorubicin administration^38, 39^. The energy sensor AMP-activated protein kinase (AMPK) is extremely sensitive to cellular AMP and ATP levels and is activated by elevated AMP/ATP ratio^40^. The increased phosphorylation of AMPK by doxorubicin suggests disruption of mitochondria, resulting in a significant depletion of cellular ATP, which may increase AMP/ATP ratio^41^. In contrast, previous studies have suggested that AMPK signaling is inhibited in the heart following doxorubicin treatment in a Langendorff rat-model^42^. The difference in results between that study and our present study may be attributed to the different models and/or differences in the dosage and regimen of doxorubicin administration. Additionally, doxorubicin can accelerate membrane lipid peroxidation, leading to the production of cytotoxic end-products, and the disruption of unsaturated fatty acyl chains^39^. These effects may be associated with the activation of AMPK, which functions to regulate the cellular lipid and glucose metabolism. To further discern the potential involvement of Cathepsin K in glycolytic changes we examined cardiac lactate levels. In organisms, the lactate is the end product of glycolysis. Under stressed condition or with circulatory dysfunction, more lactate is generated by the conversion of pyruvate, which catalyzed by the enzyme lactate dehydrogenase. Enhanced circulating and myocardial levels of lactate dehydrogenase have been recognized as a biomarker for cardiomyopathy and heart failure and is closely associated with cardiac dysfunction^43–46^. Our results revealed that doxorubicin treatment resulted in elevated lactate levels in the heart, which may suggest a decreased oxidative phosphorylation, leading to reductions in ATP production. The cardiac energy stress caused by reduced ATP after doxorubicin treatment may be compensated with higher AMPK to promote both lipid and glucose metabolism. Recent studies also suggest that AMPK activation can promote apoptosis^47^. Additionally, activation of NF-κB has been considered as an alternative mechanism leading to doxorubicin-induced apoptosis in cardiomyocytes^48^. The p65 subunit of NF-κB can cause metabolic disturbance and inflammation by PGC-1α^49^. Additionally, we found a significant increase in the pro-inflammatory cytokine IL-6 in the myocardium, which is reported to be associated with the development of heart failure^50^. In contrast, we did not observe any changes in the levels of IL-1β and TNF-α. A plausible explanation for this is that while IL-6 is induced after transient stimulation with doxorubicin, TNF-α was found to be induced at higher doses with repeated injections^51, 52^. Furthermore, decreased p-AKT induced by doxorubicin indicates a reduced survival signal in the cardiomyocytes, and is consistent with elevated levels of apoptosis^53^. Most importantly, these changes were not seen in Cathepsin K conditional knockout mice, confirming the benefit of targeting Cathepsin K to improve cardiovascular and metabolic homeostasis with anti-inflammatory and anti-apoptotic response.

To further understand the potential role of the activation of AMPK and NF-κB, as well as apoptosis in the protective effects of Cathepsin K ablation in doxorubicin-induced cardiotoxicity, we examined the effect of an AMPK activator AICAR, NF-κB inhibitor PDTC and apoptosis activator II on cardiomyocyte contractile function in the presence or absence of Cathepsin K inhibitor and doxorubicin treatment. Cathepsin K inhibitor-II restored doxorubicin-induced myocyte contractile anomalies, which was blunted by the pre-treatment of apoptosis activator II and AICAR, suggesting a role of AMPK and apoptotic signaling in the cardioprotection accorded by Cathepsin K knockout. PDTC on the other hand, partially attenuated doxorubicin-induced abnormal cardiomyocyte contractility, indicating that NF-κB activation may not be the primary mechanism involved. Further studies using knockout and overexpression of specific proteins are necessary to ascertain the role of these pathways that the cardioprotection offered by ablation of Cathepsin K.

In summary, our present study demonstrates, for the first time, that cardiac-specific deletion of Cathepsin K rescues mice from doxorubicin-induced cardiac structural and functional anomalies, which may be mediated by reconciling doxorubicin-induced imbalance of cardiac energy metabolism, NF-κB activation, and elevated apoptosis. These observations are particularly significant given the fact that Cathepsin K is upregulated in the failing heart. Inhibition of Cathepsin K could represent a therapeutic strategy in the management of cardiac dysfunction.

## Methods and materials

### Generation of cardiac *Ctsk*-CKO mice and treatments

To generate cardiac Cathepsin K knockout mice (*Ctsk*-CKO mice), three JM8 embryonic stem (ES) cell clones (HEPD0727 clones C11, F11, and H09), with conditional potential targeting exon 2–5 of *Ctsk* (Fig. 1a) were obtained from the European Mouse Mutant Cell Repository (EuMMCR). Male ES cells (C57BL/6N genetic background) were injected into C57BL/6 blastocysts at the University of Washington Transgenic Resources Program. The chimeras and the resulting germline offspring are from the C11 clone. The targeted *Ctsk* allele contains a reporter-promoter LacZ, FLP-*FRT* and Cre-*loxP* sites (Fig. 1a). Pups were genotyped for the presence of LacZ, using a LacZ TaqMan primer/probe marker set purchased from Applied Biosystems/Life Technologies and the probe Mr03987581_mr (amplicon length 77 bp) (Fig. 1d). These mice were crossed with mice expressing flippase (FLP) recombinase (B6.129S4-Gt(ROSA)26Sor<tm2(FLP*)Sor>/J (The Jackson Laboratory)^54^, resulting in offspring in which the LacZ reporter and neo cassette had been deleted (Fig. 1b). FLP positive LacZ negative mice were then crossed with those expressing Cre recombinase under control of the cardiac-specific murine alpha myosin-heavy chain (*myh6*) promoter (B6N.FVB(B6)-Tg(Myh6-cre)2182Mds/J; The Jackson Laboratory; C57BL/6 background), as shown in Fig. 1c to generate both control (Myh-Cre^−^; *Ctsk* ^fl/fl^) and cardiomyocyte-specific *Ctsk*-CKO (Myh-Cre^+^; *Ctsk* ^fl/fl^) littermate mice. Control and *Ctsk*-CKO, as well as *Ctsk* floxed mice were genotyped by PCR using FLP and Myh-Cre primers (Fig. 1e,f). The sequences of the primers are shown in Table 1. Both FLP and Myh-Cre primers were designed by the Jackson Lab. The experimental procedure was approved by the Institutional Animal Use and Care Committees of the University of Wyoming, Laramie, WY and the University of Washington, Seattle, WA). Four-month-old control and *Ctsk*-CKO mice (both male and female) weighing 25 ~ 28 g were randomly assigned to injections of doxorubicin (10 mg/kg, i.p. at 3-day intervals, 20 mg/kg cumulative) or vehicle^18^. One week after the first injection, body and tissue weight, echocardiographic properties, cardiomyocyte contractile properties, and calcium handling were evaluated. Cardiac structure was assessed by histomorphology. Myofibrillar protein markers, NF-κB signaling, and apoptosis were determined by western blot.

**Table 1:** Primer sequence for genotyping and RT-PCR

Protocol primers for FLP genotyping			Reference/note
Primer	Sequence 5′-->3′	Primer type	Designed by the Jackson Lab
olMR8545	5′-AAAGTCGCTCTGAGTTGTTAT-3′	WT and mutant forward	
oIMR8546	5′-GGAGCGGGAGAAATGGATATG-3′	WT reverse	
oIMR8502	5′-GCGAAGAGTTTGTCCTCAACC-3′	Mutant reverse	
Protocol primers for Myh-Cre genotyping			
Primer	Sequence 5′-->3′	Primer type	Designed by the Jackson Lab
oIMR8744	5′-CAAATGTTGCTTGTCTGGTG-3′	Internal positive control forward	
oIMR8745	5′-GTCAGTCGAGTGCACAGTTT-3′	Internal positive control reverse	
9543	5′-ATGACAGACAGATCCCTCCTATC-3′	Transgene forward	
9544	5′-CTCATCACTCGTTGCATCATCGA-3	Transgene reverse	
RT-PCR primers for mouse Cathepsin K and 18S			
Primer	Sequence 5′-->3′	Primer type	Designed on IDT
*Ctsk-*exon 2	5′-TCAAGTTTCTGCTGCTACC-3′	Forward	
*Ctsk-*exon 2	5′-GCTTCTGGTGAGTCTTCTTC-3′	Reverse	
*Ctsk*	5′-GGGCCAGGATGAAAGTTGTA-3′	Forward	^8^
*Ctsk*	5′-CACTGCTCTCTTCAGGGCTT-3′	Reverse	
*Rn18s*	5′-AGTGACAAGAAATAACAATACAGG-3′	Forward	
*Rn18s*	5′-CCTGCTTTAAGCACTCTAATTTTC-3′	Reverse	

### Echocardiographic assessment

Cardiac geometry and function were evaluated in anesthetized (isoflurane 1.2% administered with a calibrated vaporizer and an inhalant) mice using a two-dimensional (2D) guided M-mode echocardiography (Phillips Sonos 5500) equipped with a 15–6 MHz linear transducer (Phillips Medical Systems, Andover, MD, USA). Adequate depth of anesthesia was monitored using toe reflex. The heart was imaged in the 2D-mode in the parasternal long-axis view with a depth of 2 cm. The M-mode cursor was positioned perpendicular to interventricular septum and posterior wall of left-ventricle (LV) at the level of papillary muscles from the 2D-mode. Diastolic wall thickness, end-diastolic dimension (EDD) and end-systolic dimension (ESD) were measured. All measurements were done from leading edge to leading edge in accordance with the Guidelines of the American Society of Echocardiography. LV mass was calculated as 0.8(1.04[([LVEDD + IVSd + PWd]3−LVEDD3)]) + 0.6. LV fractional shortening was calculated as [(EDD-ESD)/EDD] × 100. Normalized LV mass was calculated as LV mass (mg)/body weight (g)^55^.

### Isolation of cardiomyocytes and the treatment

Cardiomyocytes were isolated from wild type (WT), control and conditional knockout mice were treated with doxorubicin or vehicle as described previously^56^. After ketamine/xylazine sedation, hearts were removed and perfused with Ca^2+^-free HEPES-buffered Tyrode’s solution containing (in mM): NaCl 120, KCl 15, KH_2_PO_4_ 0.6, Na_2_HPO_4_ 0.6, NaHCO_3_ 4.6, MgSO_4_ 1.2, HEPES 10, taurine 30, glucose 10, butanedione monoxime 10 at pH 7.4, and gassed with 95% oxygen and 5% carbon dioxide. Hearts were digested with 1 mg Liberase TH (Roche Diagnostics, Indianapolis, IN, USA) in 20 ml perfusion buffer for 10–15 min to collect the cells. Only rod-shaped myocytes with clear edges were selected for the study. To assess the role of AMPK and apoptosis in Cathepsin K-regulated cardiomyocyte contractile response following doxorubicin exposure, cardiomyocytes from adult WT mice were pre-treated with AMPK activator AICAR (500 µM) or apoptosis activator II (AAII, 10 µM) 30 min prior to the treatment of Cathepsin K inhibitor-II (CatK I, 1 µM) and doxorubicin (Dox, 1 µM, 30 min)^57–59^. To evaluate the role of NF-κB in response to doxorubicin, cardiomyocytes from WT mice were incubated with NF-κB-specific inhibitor PDTC (100 µM, 30 min) along with doxorubicin challenge (1 µM)^60,61^.

### Cell shortening/relengthening

Mechanical properties of cardiomyocytes were evaluated using a SoftEdge Myocam® system (IonOptix Corporation, Milton, MA). Cardiomyocytes were placed in a chamber mounted on the stage of an inverted microscope (Olympus IX-70) and superfused with a HEPES buffer containing 1 mM CaCl_2_. Myocytes were field stimulated with suprathreshold voltage at a 0.5 Hz frequency, using a pair of platinum wires placed on opposite sides of the chamber connected to an FHC stimulator (Brunswick, NE). IonOptix SoftEdge software was utilized to capture cell shortening and relengthening changes. Cell shortening and relengthening were assessed using the following indices: resting cell length, peak shortening (PS), time-to-PS (TPS), time-to-90% relengthening (TR90), and maximal velocity of shortening/relengthening (±dL/dt)^56^.

### Intracellular Ca^2+^ transient analysis

Cardiomyocytes were loaded with fura-2/AM (0.5 μM) for 15 min, and fluorescence intensity was recorded with a dual-excitation fluorescence photomultiplier system (IonOptix). Cardiomyocytes were placed onto an Olympus IX-70 inverted microscope and were imaged through a Fluor ×40 oil objective. Cells were exposed to light excited by a 75 W lamp and passed through either a 360 or a 380 nm filter, while being stimulated to contract at a frequency of 0.5 Hz. Fluorescence emissions were detected between 480 and 520 nm and the qualitative change in fura-2 fluorescence intensity (FFI) was inferred from the FFI ratio at the two wavelengths (360/380 nm). Fluorescence decay time (single exponential) was calculated as an indicator of intracellular Ca^2+^ clearance^56^.

### Myocardial histological analysis

Following anesthesia, hearts were arrested in diastole with saturated KCl, excised, segmented and immediately fixed in 10% neutral-buffered formalin at room temperature for 24 h. The specimens were then dehydrated through a series of graded alcohols, cleared in xylenes and embedded in paraffin. The serial sections were cut at 7 µm and stained with hematoxylin and eosin (H&E)^62^. Another set of sections were stained with 0.1 mg/ml Lectin-FITC conjugate (Sigma-Aldrich, L-4895) for 2 h at room temperature in the dark^63^. Slides were then washed with PBS and mounted with Fluoromount-G mounting media (Southern Biotech, Inc., Birmingham, AL).Cardiomyocyte cross-sectional area was measured and quantified from 130 random cardiomyocytes from H&E stained slides and 100 random cardiomyocytes from Lectin stained slides by using a digital microscope (×400) and the Image J (version1.39u) software. The total number of cardiomyocytes used for quantification was 230.The Masson’s trichrome staining was used to detect fibrosis in heart sections using a Masson’s Trichrome stain kit. To determine cytoplasmic vacuolization and myofibrillar loss, we quantified the data by using a scoring method. The extent of histopathology was scored on a scale of 0–3. If left ventricular heart section showed no signs of myofibrillar loss and cytoplasmic vacuolization, the score was graded as 0; slide <5% cells exhibiting early myofibrillar loss or cytosolic vacuolization was graded as 1; 5–30% cells exhibiting marked myofibrillar loss and/or cytoplasmic vacuolization was graded as 2; diffuse >30% cell damage with most exhibiting marked loss-of-contractile elements and myofibrillar disruption was graded as 3^64, 65^. There were 25 slides per group scored independently for this semi-quantification.

### TUNEL staining

TUNEL (terminal deoxynucleotidyl transferase-mediated dUTP nick-end labeling) assessment of myonuclei positive for DNA strand breaks was determined using a fluorescence detection kit (Roche Applied Science, Indianapolis, IN) and fluorescence microscopy. After dewax and rehydrate, paraffin tissue sections were permeabilized with 0.1% Triton X-100 in 0.1% sodium citrate for 8 min on ice. TUNEL reaction mixture containing terminal deoxynucleotidyl transferase (TdT), fluorescein-dUTP was added to the sections in 50-µl drops and incubated for 60 min at 37 °C in a humidified chamber in the dark. The sections were rinsed three times in PBS for 5 min each. Following embedding, sections were visualized with an Olympus DP80 microscope equipped with an Olympus MaguaFire SP digital camera. DNase I and label solution were used as positive and negative controls. To determine the percentage of apoptotic cells, the TUNEL-positive nuclei and TUNEL-negative cells were counted using the Image-Pro image analysis software (Media Cybernetics, Bethesda, MD)^62^.

### Cardiac lactate measurement

The cardiac lactate production was determined by using a lactate assay kit (Cell Biolabs, CA, USA). The procedure was performed per the product manual.

### Primer design, total RNA extraction, cDNA synthesis, reverse transcription, and real-time polymerase chain reaction

Total RNA was isolated from left ventricles using the TRIzol reagent (Invitrogen), followed by DNase digestion to eliminate genomic DNA contamination^66^. RNAs were quantified using a NanoDrop^TM^ 2000 spectrophotometer (Thermo Fisher Scientific). Synthesis of cDNA and reverse transcription was performed using 1 µg total RNA in a 20-µl system following the instructions of iScript^TM^ cDNA synthesis kit (Bio-Rad). *Ctsk* primers located in exon 2 for qPCR were designed by using the PrimerQuest tool on Integrated DNA Technologies’ (IDT) website (http://www.idtdna.com/primerquest/home/index). The quantitative real-time PCR was performed for *Ctsk*, *Ctsk*-exon 2, and *Rn18s* (housekeeping gene) using a C1000 Touch Thermal Cycler CFX96^TM^ Real-Time System (Bio-Rad) per the iQ^TM^ SYBR^®^ Green Supermix (Bio-Rad) instructions. Real-time PCR was triplicated for each cDNA sample. The primer sequences are shown in Table 1.

### Western blot analysis

Protein samples were prepared as described previously and separated on SDS-polyacrylamide gels, and transferred electrophoretically to nitrocellulose membranes^55^. The membranes were blocked with 5% milk in TBS-T, and were incubated overnight at 4 °C with anti-Cathepsin K (1:500; ab19027), anti-desmin (1:1000; CST5332), anti-sarcomeric α-actinin (1:1000; ab9465), anti-NFκB-p65 (1:1000; CST8242), anti-phospho-IκBα (Ser32/36, 1:500; CST9246), anti- IκBα (1:1000; CST4812), anti-interleukin-1β (IL-1β, 1:500; CST8689), anti- anti-interleukin-6 (IL-6, 1:500; CST12912), anti-TNF-α (1:500, CST3707), anti-phospho-AMPK (Thr172, 1:1000; CST2535), anti-phospho-AKT (Ser473,1:1000; CST4060), anti-cleaved caspase-3 (1:500; CST9661), anti-Bcl-2 (1:1000; CST2870), anti-BAX (1:1000; CST2772), anti-GAPDH (loading controls, 1:1000; CST2118) and anti-α-tubulin (loading controls, 1:1000; CST2144) antibodies. Blots were incubated with horseradish peroxidase (HRP)-conjugated secondary antibody (1:3000; CST7074, CST7076). Antigens were detected by the luminescence method. Band densities were determined using Quantity One software (Bio-Rad, version 4.4.0, ChemiDoc XRS).

### Statistical analyses

The data were presented as mean ± SEM. Statistical significance (*p* < 0.05) for each variable was estimated by an unpaired *t*-test (two-tailed) or a one-way analysis of variance (ANOVA) followed by a Tukey’s post hoc analysis.

## Electronic supplementary material

Supplemental Material
