# Correction: Cardiomyocyte-specific disruption of Cathepsin K protects against doxorubicin-induced cardiotoxicity

**DOI:** 10.1038/s41419-019-2113-0

**Published:** 2019-12-05

**Authors:** Rui Guo, Yinan Hua, Jun Ren, Karin E. Bornfeldt, Sreejayan Nair

**Affiliations:** 1 Center for Cardiovascular Research and Alternative Medicine, School of Pharmacy College of Health Sciences, University of Wyoming, Laramie, WY 82071 USA; 2 UW Medicine Diabetes Institute, Departments of Medicine, Division of Metabolism, Endocrinology and Nutrition, and Pathology, School of Medicine, University of Washington, Seattle, WA 98109 USA

**Correction to: Cell Death & Disease**

10.1038/s41419-018-0727-2 published online 07 June 2018

Since online publication of this article, the authors noticed that there was an error in the TUNEL staining images used to compile Fig. 8a. The error had no impact on the results of the study. The corrected image is provided below. The authors apologise for any inconvenience caused.

**Figure:**
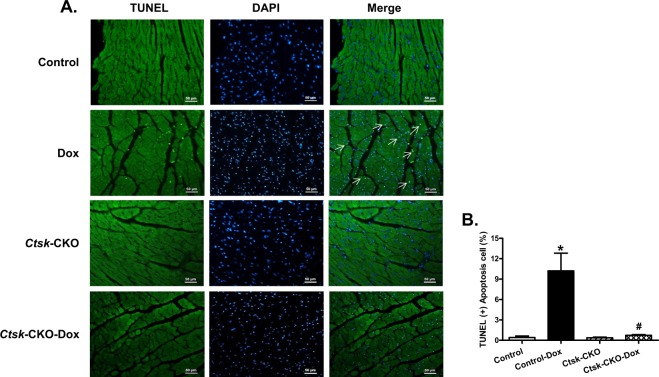
Fig. 8

