# Supplementary material for: Cardiomyocyte-specific disruption of Cathepsin K protects against doxorubicin-induced cardiotoxicity
Source: Cell Death Dis. 2018 Jun 7;9(6):692. doi: 10.1038/s41419-018-0727-2 (PMC5992138; doi:10.1038/s41419-018-0727-2)
Supplement: Supplementary file 1 — Supplemental Material [file 41419_2018_727_MOESM1_ESM.docx]

**Cardiomyocyte-Specific Deletion of Cathepsin K Protects Against Doxorubicin-Induced Cardiac Dysfunction and Structural Abnormality**

Rui Guo^1^, Yinan Hua^1^, Jun Ren^1^, Karin E. Bornfeldt^2^ and Sreejayan Nair^1^

^1^Center for Cardiovascular Research and Alternative Medicine, School of Pharmacy College of Health Sciences, University of Wyoming, Laramie, WY 82071, USA

^2^UW Diabetes Institute, Departments of Medicine, Division of Metabolism, Endocrinology and Nutrition, and Pathology, School of Medicine, University of Washington, Seattle, WA 98109, USA

**
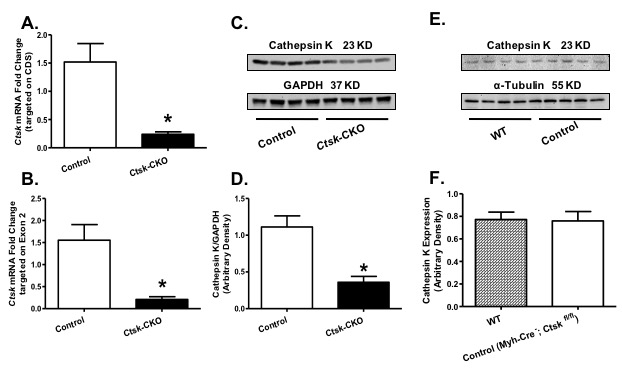
**

**Supplementary Figure 1.** Cathepsin K expression in the mouse heart. **A.** *Ctsk* mRNA fold change in control and *Ctsk*-CKO groups; **B.** *Ctsk* mRNA fold change targeted on exon 2 in control and *Ctsk*-CKO groups; **C.** Representative bands of cathepsin K and GAPDH in control and *Ctsk*-CKO groups; **D.** Cathepsin K protein expression normalized to GAPDH in control and *Ctsk*-CKO groups. **E.** Representative bands of cathepsin K and α-Tubulin in WT and control groups; **F.** Cathepsin K protein expression normalized to α-Tubulin in in WT and control groups. Mean ± SEM, n= 5–9 mice per group. *p<0.05 vs. Control group.

**B.**

**A.**

**Supplementary Figure 2.** Body weight gain and heart weight from control and cardiac specific *Ctsk*-CKO mice treated with or without doxorubicin. **A.** heart weight; **B.** Body weight gain. Mean ± SEM, n = 6–8 mice per group, *p<0.05 vs. Control group, #p<0.05 vs. Control-Dox group.

**Supplementary Figure 3.** Cardiac lactate level from control and cardiac specific *Ctsk*-CKO mice treated with or without doxorubicin. Mean ± SEM, n = 3–4 mice per group, *p<0.05 vs. Control group, #p<0.05 vs. Control-Dox group.


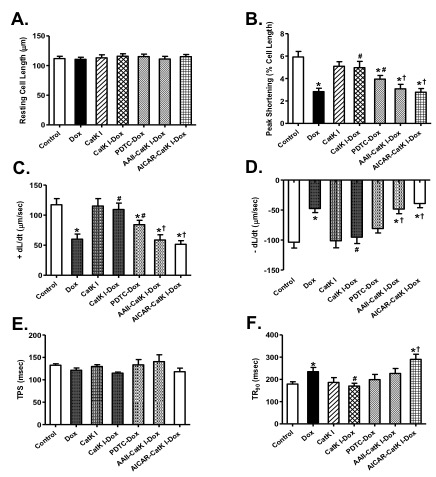


**Supplementary Figure 4.** Effect of NF-κB specific inhibitor PDTC, apoptosis activator-II and AMPK activator AICAR on cardiomyocyte contractile properties in the absence or presence of doxorubicin or cathepsin K inhibitor. A. Resting cell length; B. peak shortening (PS), normalized to cell length; C. maximal velocity of shortening (+dL/dt); D. maximal velocity of relengthening (- dL/dt); E. time-to PS (TPS); F. time-to-90% relengthening (TR90). Mean ± SEM, n=42–53 cells from three mice per group. *p<0.05 vs. Control group, #p<0.05 vs. Dox group, †p<0.05 vs. CatK I-Dox group.
